# Medical and Physical Intelligence

**Published:** 1800-08

**Authors:** 


### MEDICAL AND PHYSICAL INTELLIGENCE.

Prof. WURZER, of Bonn, relates a caſe of venereal diſeaſe, where nitric acid was employed with the greateſt ſucceſs. A young man being apparently cured of local venereal affections, had ſoon after venereal ulcerations in the mouth, and at the ſame time, an exanthematous eruption breaking out over the body, particularly in the face. After having undergone ſix ſalivations, the chancres healed, but the eruption, which covered the whole body, continued to increaſe, and began to ulcerate at different parts of the body. His conſtitution was ſo extremely debilitated, that Prof. W. to whom he applied for advice, was obliged to ſtrengthen him by a proper diet and regimen and corroborant remedies, combined with ſuch as are recommended in ſimilar caſes, as guaiacum, ſarſaparilla, &c. Although his ſtrength was ſomewhat re-eſtabliſhed, yet no change was brought on in the eruptions and ulcerations, and Prof W. began now to give him thoſe mercurials which are leſs apt to produce ſalivation; as, ſublimate and mercurius cinerus, or ſolubilis Hahnemann; but after taking only one or two doſes, a vehement ſalivation came on, which again weakened him ſo much, that he was obliged to leave off taking mercurials, particularly as the ſymptoms increaſed, and moſt excruciating pains of the bones (dolores oſteocopi) were now produced, of which he felt little before.— Under theſe circumſtances, Prof. W. had recourſe to Nitric Acid, of which he gave him one drachm diluted with two pounds of water, to be taken in twenty-four hours, with a decoction of ſarſaparilla. Having continued this for ſome time, the eruptions and ulcerations began to dry, the pains in the bones ceaſed by degrees, and in the ſpace of three weeks the patient was quite recovered; by ſtrengthening medicines and cold baths his health was entirely re-eſtabliſhed. Prof. Hufeland, in whoſe Practical Journal, Vol. iv. No. 19, this caſe is related, remarks, that nitric acid, according to his experience, ſometimes proved of great benefit in affections which are rather conſequences and morbid remainders of venereal diſeaſe, and not any longer depending on the poiſon; but that he never ſaw it of any uſe in a true venereal complaint, while the poiſon was ſtill active.

---

Dr. HARGENS, of Kiel, has made ſome obſervations, which ſeem to confirm the great uſe of alkali in convulſive affections.— A child, three years of age, was vehemently afflicted with convulſive fits, particularly in the left ſide; againſt which, flores zinci, muſk, extractum hyoſciami, clyſters of aſſafœtida, anodyne inunctions with opium, and bliſters, were all applied in vain. The oleum tartari per deliquium, or aqua kali preparati, was now given to twenty-five drops every five minutes. Immediately after the fir doſe, th convulſions diminiſhed; and after the ſecond, their vio lence h d entirely ceaſed. —— Another child, nine months old which ſuffered dreadful convulſions, took fifteen drops every five minutes, and was almoſt inſtantly relieved by it. It proved likewiſe of very great ſervice in other ſpaſmodic affections; and, at leaſt, as a palliative remedy in cardialgic paroxyſms, particularly thoſe of the hyſteric kind, and in chlorotic conſtitutions. *(Hufeland's Practical Journal, Vol. viii. No.* 1.*)*

---

Dr. Gebel relates an inſtance of the great uſe of the extractum aconiti in a vehement rheumatic cardialgy, given in a doſe of two grains every morning and evening, after which it ceaſed immediately, though large doſes of opium had been applied before without any effect. This remedy, which ſeems to be neglected in this country, has ſtill kept its reputation amongſt the phyſicians of Germany, principally in obſtinate rheumatic complaints. *(Ibid.)*

---

In the ſame Number of that Journal, we find the uſe of Mercurius ſublimatus corroſivus, very much recommended in obſtinate amauroſis, and confirmed by two caſes. The firſt was a patient much affected with rheumatiſm, of which he was relieved by guaicum, aconitum, ſulphur, antimonials, &c.; but the amauroſis ſuffered no change by theſe remedies, and rather got worſe. After having tried oleum cajeput, valerian, electricity, &c. without effect, the patient was ordered to take the corroſive mercury in the following way:—℞. *Mercurii ſublimat. corroſiv. gr. ij. ſolve in aether vitriol.* ℥*ij.* of which to take 10 drops every morning and evening in half a cup of warm milk. When he had uſed about 12 grains of mercury, the ſight was ſo far re-eſtabliſhed, that he was able to read and write. Though he was perfectly well, it was thought proper to continue the mercury for ſome time; but as the ſight ſeemed to decreaſe by this continuation, it was left off. It is remarked by farther experience, that probably as ſoon as a certain doſe has had its effects, farther benefit is not to be expected from the continuation of this remedy, but rather the contrary. Another patient, quite blind of both eyes, uſed it for ſix weeks, in which time he entirely recovered the ſight of the left eye, though no change was produced in the right; but it was not thought proper to continue the remedy on the above account. It is, however, proper to obſerve, that this powerful remedy ſhould be uſed with caution: it ſeems particularly indicated, when any ſuſpicion of a venereal cauſe exiſts, or in rheumatic amauroſes, as it has been lately recommended in ſuch complaints. *(Ibid.)*

---

In the ſame Journal the following fact is mentioned, which deſerves the attention of our readers.—An anatomical preparation of indurated glands from a very ſcrophulous ſubject being put into ſpiritus vini, was found ſome time after to be incruſted with ſaline cryſtals. To prove that they really came from the preparation, and were were not contained in the ſpirit, it was laid into other ſpiritus vini wherein likewiſe cryſtals appeared upon it, but none upon another preparation, which was put into the former ſpirit. By a chemical analyſis, thoſe cryſtals were found to conſiſt of ſaccharic acid. This inſtance ſhews what changes are produced in the chemical mixture of the humours of the body by the morbid affections of it, and ſeems to prove, that a particular corruption of the lymph, of an acid nature, takes place in the ſcrophula, which, though originating from a vitiated action of the lymphatic ſyſtem, muſt be conſidered as the prime cauſe of many otherwiſe inexplicable ſymptoms of that diſeaſe, and which ſeems, at the ſame time, to explain the uſe of abſorbent and alkaline remedies here. (*Ibid.* *vol.* vii. *No.* 1.)

---

Citizens Buniva and Vauquelin have, in the analyſis of the liquor ammii of a cow, diſcovered that it contains a peculiar acid, which they call *acide amniotique.* We ſhall, perhaps, communicate to our readers, the paper on this ſubject, which is printed in the *Annales de Chemie, Ventôſe, An. viii.*

---

We have already mentioned in one of the Numbers of this Journal, that the late Mr. Girtanner, of *Gottingen,* meant to have diſcovered the analyſis of the azote. In a letter to Cit. *Van Mons,* he gives a ſhort account of it. Azote is now, according to him, a compound ſubſtance conſiſting of hydrogen and oxygen in the proportion of 0,21 of the former, and 0,79 of the latter; and conſequently, nothing but an oxyde of hydrogen. Our eudiometrical operations are erroneous with reſpect to the azote, becauſe this is but a production, and did not exiſt in the air before the experiments were made. Alum and aluminous earth are the ſubſtances which moſt eaſily change atmoſpheric air into azote. It is produced by reſpiration, the oxygen, of which a part is always condenſated in the lungs, combining itſelf with the hydrogen, and changing it into azote; which however, more viſibly takes place by bringing hydrogen in contact with wet aluminous earth. We have to expect a treatiſe upon the ſubject. (*Ibid.*)

---

Mr. Eckeberg gives a more accurate account of a blackiſh ſtone found at Ytterby in Sweden, and of the new earth contained therein, called Yttererarth. This foſſil was firſt analyſed by Mr. Gadolin, but he does not mention the feld ſpar which Mr. Eckeberg found mixed in it. From 100 parts of the foſſil he obtained 47 ½ parts of that new earth. It is ſoluble in all acids, to which it imparts a ſweetiſh taſte, cauſtic alkalis have no effect upon it. It forms with ſulphuric acid an eaſily ſoluble ſalt, which ſoon ſhoots into cryſtals, and keeps its acid entire. (*Swediſh Tranſactions, Vol. xviii.* 1797.)

---

Prof. Parrot, of Riga, has made ſeveral experiments, by which he attempts to ſhow, that coal is a compound ſubſtance, that that carbonic acid is only contained accidentally in it, as in chalk and lime; that the true carbon differs from it, and is either nothing but hydrogen, or a combination of hydrogen and azote, and consequently that there exifts no proper hydrogen, but only carbon, and that water is now compofed of carbon and oxygen. *(Vorgt's Magazin for Natural Science, Vol. ii. No. 1, p. 131.)*

---

Dr. Seybert, of Philadelphia, writes in a letter to Prof. Blumenbach of Gottingen, that the Philofophical Society of Philadelphia has now obtained a large collection of bones from the weftern country, of that prodigious unknown animal, called *Mammut Ohioticum,* which will throw a new light upon its olteology and form. It is remarkable, that fome bones of the pofterior extremities have a great likenefs to human bones, as the patella, tibia, the tali calcaneus, and the os cubiforme. *(Ibid.)*

---

Prof. Linck, of Boftock, relates in a letter to the fame Profeffor, a rare inftance of a mule having become pregnant in the province of Algarbia, in Portugal, where it was fhown for money as a great curiofity, firft pregnant, afterwards with the young one. Inftances of mules propagating their own fpecies are extremely rare; however, they can be ufed as a ftrong argument againft a notion adopted by Ray and Buffon, that thofe animals which are capable of producing with one another young ones, and are poffeffed of fecundity, belong to one and the fame fpecies; becaufe, if this was a general rule, the horfe and afs ought, according to thofe rare inftances, to be confidered as of the fame fpecies, however different they are in their external and internal ftructure. *(Ibid.)*

---

Dr. Meyer, of Berlin, relates an experiment, by which he was convinced that iron particles entered into the blood with the chyle. He gave at firft to a dog five grains of limatura martis per day, and afterwards the fame portion in the morning and evening. Having continued this for eight days, the dog was killed, an hour and a half after he had taken the laft dofe. A ligature being applied to the ductus thoracicus, Dr. M. obtained from it about one drachm of chyle, in which not the leaft iron particle could be traced, either by any reagency or by combuftion. All the fluid found in the ftomach and inteftines, fhewed that it contained iron, by a black precipitation, when mixed with a folution of volatile liver of fulphur. To be ftill more fure of the non-exiftence of iron in the chyle, in cafe it fhould be prefent in fuch minute portion as not to be affected by reagency, he mixed a fmall quantity of a very diluted folution of vitriolum martis with the chyle, and tried it with the volatile liver of fulphur, when a black precipitation was immediately produced. *Reil's Archives for Phyfiology, Vol. iv. No. 3.*

---

Dr.

Dr. Schmidt, of Ofnabruck, who has related that curious inftance of abftinence from food and drink, which we communicated to our readers in the laft Number of this Journal, acquaints the public, that the whole cafe is difcovered to be a moft fubtle impofition, whofe proceeding and difcovery he is going to publifh in a pamphlet; and we fhall not omit to give an account of it here, as a circumftance very interefting with refpect to Pfichology, and alfo as an inftance how cautious phyficians always ought to be in fimilar cafes, and how little the moft appearing circumftances are to be trufted.

---

Mr. Pasumot, mentions in his Voyages Phyfiques dans les Pyrénées, 1788 and 1789, Paris, 1797, that on his journey to Gavarin, he has heard of a family being extinguifhed at Vifos, about feventeen years ago, whofe members were all eight feet high, and amongft them a man reached the age of 110 years.

---

Dr. Wolcot, (the admired fatirift, Peter Pindar) has it in contemplation to publifh a Treatife on the general Caufes of Deafnefs, with the modes of cure. Such a publication is certainly a *defideratum*, as no minute and fatisfactory account of the complaints of the Ear has hitherto made its appearance.

---

Many unfounded reports having been circulated, which have a tendency to prejudice the mind of the Public againft the Inoculation of the Cow-Pox; we, the underfigned Phyficians and Surgeons, think it our duty to declare our opinion, that thofe perfons who have had the Cow-Pox, are perfectly fecure from the infection of the Small Pox, provided fuch infection does not exift in the fyftem at the time of the inoculation for the Cow-Pox.

We alfo declare, that the inoculated Cow-Pox is a much milder and fafer difeafe than the inoculated Small-Pox.

William Saunders, M. D.
Matthew Baillie, M. D.
Henry Vaughan, M. D.
Maxwell Garthfhore, M. D.
John Coakley Lettfom, M. D.
James Sims, M. D.
John Sims, M. D.
William Lifter, M. D.
Robert Willan, M. D.
C. Stanger, M. D.
Alexander Crichton, M. D.
Thomas Bradley, M. D.
Thomas Denman, M. D.
John Squire, M. D.
Richard Croft, M. D.
Robert Batty, M. D.
R. J. Thornton, M. D.
Richard Dennifon, M. D.
Henry Cline.
Edward Ford.
Aftley Cooper.
John Abernethy.
Jofeph Hurlock.
William Blair.
Samuel Chilver.
J. M. Good.
James Horsford.
Francis Knight.
James Leighton.
James Moore.
Thomas Paytherus.
Thomas Pole.
J. W. Phipps.
John Ring.
James Simpfon,
H. L. Thomas.
Jonathan Wathen.
Thomas Whateley.

